# Not Your Usual Exposure: Tuberculosis Contact Investigation Related to Contaminated Bone Allograft

**DOI:** 10.1017/ash.2024.285

**Published:** 2024-09-16

**Authors:** Christy Scipione, Laraine Washer, Emily Stoneman, Amanda Valyko, Jennifer Sweeney

**Affiliations:** University of Michigan Health; University of Michigan; Michigan Medicine

## Abstract

**Background:** Mycobacterium tuberculosis transmission through contaminated bone allograft product is unusual and was first described in 2021 with a second outbreak in 2023. In July 2023, Michigan Medicine conducted contact tracing for healthcare personnel (HCP), patients, and visitors following exposure to an immunocompromised patient with surgical site infection and subsequent widely disseminated tuberculosis (bacteremia, pulmonary, lymphadenopathy) following spinal fusion with bone allograft in April 2023. The patient was in the emergency department, operating room (OR), and inpatient units for 9 days prior to initiation of Airborne Precautions (AP). **Methods:** Michigan Medicine is a 1,107 bed academic hospital. HCP are screened for tuberculosis with interferon-gamma release assay (IGRA) testing upon hire and following tuberculosis exposure. Exposure testing includes baseline IGRA testing and follow-up testing at 10-12 weeks post exposure. Exposure criteria for this investigation was defined as sharing room airspace with the tuberculosis patient prior to initiation of airborne precautions or Central Sterile Processing Department (CSPD) staff involved with instrument decontamination without the use of a respirator. Of note, universal masking with surgical masks was not required during this time for staff and patients/visitors, with the exception of CSPD and OR staff. Contact tracing was performed by Infection Prevention and Occupational Health Services managed all test results and conversions. **Results:** 176 employees from perioperative care areas (n=30), CSPD (n=7), OR (n=9) and inpatient units (n=130) were IGRA tested. Five employee conversions were identified: one surgeon, one circulating OR nurse, two CSPD decontamination staff, and one respiratory therapist. At time of detection, none of the conversions had evidence of active tuberculosis. Additionally, 46 patients and visitors were tested with zero conversions. HCP compliance with IGRA testing was initially 15% before engagement from hospital and unit leadership and human resources. With intervention, employee compliance reached 100%. **Conclusion:** Despite standard use of surgical masks for OR and CSPD staff, aerosolization of infected bone graft material played an important role in tuberculosis transmission during surgery and instrument cleaning. Respiratory therapy practices in the ICU setting likely also increased risk for pulmonary tuberculosis transmission. Achieving 100% HCP compliance for baseline and follow-up IGRA testing is challenging and requires engagement of both unit and hospital leadership and human resources to ensure all HCP are tested.

